# MDMA and memory, addiction, and depression: dose-effect analysis

**DOI:** 10.1007/s00213-022-06086-9

**Published:** 2022-02-18

**Authors:** Madeline M. Pantoni, Jinah L. Kim, Kaitlin R. Van Alstyne, Stephan G. Anagnostaras

**Affiliations:** 1 Molecular Cognition Laboratory, Department of Psychology, University of California San Diego, La Jolla, CA USA; 2 Translational Psychedelic Research Program, Department of Psychiatry and Behavioral Sciences, University of California San Francisco, CA San Francisco, USA; 3 Molecular Cognition Laboratory, Program in Neurosciences, University of California San Diego, La Jolla, CA USA

**Keywords:** MDMA, 3,4-Methylenedioxymethamphetamine, Mice, Memory, Addiction, Depression, Behavioral sensitization, Conditioned place preference, Pavlovian fear conditioning, Stimulant

## Abstract

**Rationale:**

±3,4-Methylenedioxymethamphetamine (MDMA) is a recreational drug that shows substantial promise as a psychotherapeutic agent. Still, there is some concern regarding its behavioral toxicity, and its dose-effect relationship is poorly understood. We previously explored the role of dose in the cognitive effects of MDMA in a systematic review of existing literature and found no evidence in animals that MDMA impairs memory at low doses (< 3 mg/kg) but mixed results at high doses (≥ 3 mg/kg). Since this review comprised mostly of single-dose studies and an assortment of methodologies, an empirical dose-ranging study on this topic is warranted.

**Objectives:**

The current study aims to evaluate the conclusion from our systematic review that 3 mg/kg may be the threshold for MDMA-induced amnesia, and to further understand the dose-effect relationship of MDMA on behavioral assays of memory, addiction, and depression.

**Methods:**

We systematically examined the effects of 0.01 to 10 mg/kg MDMA on Pavlovian fear conditioning; behavioral sensitization, conditioned place preference, and conditioned responding; and the Porsolt forced swim test in mice.

**Results:**

High doses of MDMA (≥ 3 mg/kg) produced amnesia of fear conditioning memory, some evidence of an addictive potential, and antidepressant effects, while low doses of MDMA (≤ 1 mg/kg) had no effect on these behaviors.

**Conclusions:**

The present dose-ranging study provides further evidence that 3 mg/kg is the threshold for MDMA-induced amnesia. These findings, in addition to our systematic review, demonstrate that careful selection of MDMA dose is critical. High doses (≥ 3 mg/kg) should likely be avoided due to evidence that they can produce amnesia and addiction. Conversely, there is little evidence to suggest that low doses, which are usually administered in clinical studies (approximately 1–2 mg/kg), will lead to these same adverse effects. Ultra-low doses (< 1 mg/kg) are likely even safer and should be investigated for therapeutic effects in future studies.

**Supplementary Information:**

The online version contains supplementary material available at 10.1007/s00213-022-06086-9.

## Introduction

±3,4-Methylenedioxymethamphetamine (MDMA) is a widely used recreational drug that shows substantial promise as a psychotherapeutic agent (Feduccia et al. 2018; Sessa and Nutt 2015; UNODC 2020). MDMA targets various brain receptors and transporters with marked and preferential effects on the serotonergic system; it increases extracellular levels of serotonin (5-HT), norepinephrine (NE), and dopamine (DA) by reversing their transporters (SERT, NET, and DAT) and also exhibits some affinity for 5-HT, DA, muscarinic, histamine, and adrenergic receptors (Battaglia et al. 1988; Rothman et al. 2001; Rudnick and Wall 1992; Shulgin 1986; Torres et al. 2003). MDMA is classified chemically as a methamphetamine derivative, but behaviorally it is considered a stimulant-psychedelic by its detractors and an empathogen-entactogen by its proponents (Liechti 2015; Nichols 1986). It is these latter behavioral effects—increased empathy, trust, extroversion, and sociality (collectively referred to here as “prosocial”)—that distinguish MDMA from psychostimulants and hallucinogens and are of particular interest (Bershad et al. 2016; Dolder et al. 2018; Holze et al. 2020; Hysek et al. 2014; Kamilar-Britt and Bedi 2015; Liechti 2015; Nichols 1986; Schmid et al. 2014). Given these prosocial effects, MDMA has the potential to enhance the effectiveness of psychotherapy for psychiatric conditions such as social anxiety and autism spectrum disorders (Danforth et al. 2018) or even improve social behavior as a stand-alone treatment (Heifets and Malenka 2016). Recent phase 2 and 3 clinical studies also reveal that MDMA-assisted psychotherapy is an effective therapeutic for treatment-resistant post-traumatic stress disorder (Bouso et al. 2008; Mitchell et al. 2021; Mithoefer et al. 2011, 2013, 2018; Oehen et al. 2013; Ot’alora et al. 2018) that may outperform approved pharmacotherapies (i.e., the selective serotonin reuptake inhibitors paroxetine and sertraline) in terms of efficacy (Feduccia et al. 2019).

Despite MDMA’s apparent therapeutic promise, there is some concern regarding its behavioral toxicity (Schenk and Newcombe 2018), such as the potential to elicit memory impairments, addiction, and depressed mood. Moreover, it has been widely noted that a major hindrance to the development of psychedelics for therapeutic use is the lack of dose response data (for example, see Sellers et al. 2018). This data is critical to determine which doses of a therapeutic drug may be safe and effective. For example, psychostimulants (e.g., amphetamine, methylphenidate, cocaine, modafinil) are highly effective cognitive enhancers at ultra-low and low doses but can be addictive and cognitively impairing at high doses (for review, see Wood et al. 2014). We previously explored the role of dose in the cognitive effects of MDMA in a systematic review of existing literature (Pantoni and Anagnostaras 2019) and found no evidence in animals that MDMA impairs memory at low doses (< 3 mg/kg) but mixed results regarding cognitive effects at high doses (≥ 3 mg/kg). Since this review comprised mostly of single-dose studies and an assortment of methodologies, an empirical dose-ranging study on this topic is warranted. The current study aims to evaluate the conclusion from our systematic review that 3 mg/kg may be the threshold for MDMA-induced amnesia, and to further understand the dose-effect relationship of MDMA on behavioral assays of memory, addiction, and depression.

We have generally argued that doses should be scaled between animals and humans directly by body weight unless specific evidence (e.g., actual exposure data) justifies some specific kind of alternative scaling (see Carmack et al. 2014; Pantoni and Anagnostaras 2019, and Wood et al. 2014). Low-dose MDMA (about 1 to 2 mg/kg) produces equivalent increases in plasma drug concentration and monoamine release in humans (oral administration) and rodents (parenteral administration) (Baumann et al. 2007; Green et al. 2012), but time of peak drug exposure is shorter in rodents (10 to 45 min; Baumann et al. 2009) than in humans (about 145 min; Kolbrich et al. 2008). This data justifies temporal scaling but not dose scaling between rodent and human MDMA studies (for further discussion, see Pantoni and Anagnostaras 2019). Here, we systematically examine the effects of 0.01 to 10 mg/kg MDMA on Pavlovian fear conditioning; behavioral sensitization, conditioned place preference, and conditioned responding; and the Porsolt forced swim test in mice. This range captures doses from one-tenth to ten times those used in recent clinical studies (approximately 1–2 mg/kg MDMA; Bouso et al. 2008; Danforth et al. 2018; Mithoefer et al. 2011, 2013, 2018; Oehen et al. 2013; Ot’alora et al. 2018).

Pavlovian fear conditioning is a simple and efficient tool for modeling drug effects on learning and memory in rodents (Anagnostaras et al. 2000, 2010; Carmack et al. 2014; Maren 2001). In this task, an initially neutral conditioned stimulus (CS; e.g., a tone or an environmental context) is paired with an aversive unconditioned stimulus (US; e.g., a footshock). When learning occurs as a result of this pairing, either CS alone will elicit a conditioned response (CR; e.g., fear). In rodents, fear memory is typically quantified by measuring freezing behavior in response to a CS. Both context and tone fear memory are amygdala-dependent while contextual fear memory is also hippocampus-dependent (Anagnostaras et al. 1999, 2000, 2001, 2010; Gale et al. 2004; Maren et al. 1998). Psychostimulants modulate fear learning and memory dose-dependently: they enhance long-term memory at low, clinically relevant doses (0.005–0.05 mg/kg d-amphetamine; 0.01 and 1 mg/kg methylphenidate; 0.1 mg/kg cocaine; 0.75 mg/kg modafinil) but impair long-term memory at high, abused doses (4 and 8 mg/kg d-amphetamine; 10 mg/kg methylphenidate; 15 mg/kg cocaine; 75 mg/kg modafinil) (Carmack et al. 2014; Shuman et al. 2009; Wood and Anagnostaras 2009; Wood et al. 2007). Citalopram, a highly selective serotonin reuptake inhibitor, also impairs fear memory at high doses (10 mg/kg) but has no effect at low doses (0.01–1 mg/kg) (Carmack et al. 2014). Additional evidence suggests that psychostimulant-induced memory enhancement requires the combination of both DAT and NET inhibition (see Carmack et al. 2014; Pantoni et al. 2020).

Behavioral sensitization, conditioned place preference, and conditioned responding are behaviors that reflect the addictive potential^1^ of a drug (Anagnostaras and Robinson 1996; Anagnostaras et al. 2002; Carmack et al. 2017; Robinson and Berridge 1993, 2003, 2008). Behavioral sensitization is a progressive increase in response following repeated administration of a drug and reflects dopamine system hyperactivation. Conditioned place preference is the preference for a context that has been paired with a drug and models the rewarding effects of a drug, as well as drug seeking. Conditioned responding after repeated environment-drug (CS-US) pairings is a drug-like CR to a drug-paired context and models associative learning thought to elicit craving. The effects of psychostimulants on these behaviors are also dose-dependent: low, memory-enhancing doses (0.005 mg/kg d-amphetamine; 1 mg/kg methylphenidate; 0.15 mg/kg cocaine; 0.75 mg/kg modafinil) show no evidence of sensitization or place preference while high, memory-impairing doses (1.5 mg/kg d-amphetamine; 10 mg/kg methylphenidate; 15 mg/kg cocaine; 75 mg/kg modafinil) show evidence of a high addictive potential (Carmack et al. 2014; Shuman et al. 2012). The action of high-dose psychostimulants at DAT and the ensuing increase in extracellular DA levels are largely responsible for the addictive potential of psychostimulants (Koob and Volkow 2010; Volkow et al. 1999, 2002); however, evidence suggests that drugs with weak activity at DAT (e.g., low affinity such as bupropion, or low dose such as Adderall) are not likely to produce addiction (Carmack et al. 2014; Pantoni et al. 2020).

The forced swim test is a model of depressive-like behavior and is used to screen for antidepressant drugs in rodents (Porsolt et al. 1977). In this test, animals are placed into a tank filled with water and time spent mobile (i.e., animal is active as it attempts to escape the stressful environment) versus immobile (i.e., “behavioral despair,” animal is passive as it loses hope to escape the stressful environment) is measured. Common antidepressants decrease immobility behavior in the forced swim test (Cryan et al. 2005a; Petit-Demouliere et al. 2005).

Published studies of MDMA’s effects on these behavioral tasks are primarily limited to high-dose experiments; low-dose and dose-ranging studies are lacking. On related contextual fear conditioning paradigms, rodents treated with 10 or 20 mg/kg then tested off-drug exhibited no effects (Shortall et al. 2013) or fear memory impairments (Johansson et al. 2015), respectively. On the forced swim test, rodents treated with 4 to 20 mg/kg then tested off-drug exhibited decreases (Majumder et al. 2011), increases (McGregor et al. 2003; Renoir et al. 2008; Shih et al. 2016, 2019; Thompson et al. 2004), or no changes (Abad et al. 2014; Clemens et al. 2005, 2007; Durkin et al. 2008; Ho et al. 2004) in depressive-like behavior. MDMA reliably produces behavioral sensitization in rodents treated with doses of 2 to 40 mg/kg (for example, see Åberg et al. 2007; Itzhak et al. 2003; Kalivas et al. 1998; McCreary et al. 1999; Spanos and Yamamoto 1989), and has been shown to produced conditioned responding in rodents treated with 5 mg/kg (Ciudad-Roberts et al. 2013; Gold and Koob 1989) but not in rodents treated with 3 mg/kg (McCreary et al. 1999) or 10 mg/kg (Anderson and Itzhak 2003; Varela et al. 2011). While the present study is the first to examine the effects of low-dose MDMA (≤ 1 mg/kg) on the aforementioned tasks, there are a few published dose-ranging studies on MDMA-induced conditioned place preference. Robledo et al. (2004) observed a significant conditioned place preference after repeated MDMA treatment in mice at a dose of 10 mg/kg but not at 0.3, 1, or 3.3 mg/kg. Similarly, Salzmann et al. (2003) observed a significant conditioned place preference after repeated MDMA treatment in mice at a dose of 9 mg/kg but not at 1 or 3 mg/kg. Still, in the present study, we analyze and compare the dose-effect relationship of MDMA across several behavioral tasks, utilizing the same methods as our previous psychostimulant studies (Carmack et al. 2014).

## Methods

### Subjects

A total of 184 hybrid C57BL/6Jx129S1/SvImJ (129B6; Jackson Laboratory, West Sacramento, CA, USA) male (*n* = 91) and female (*n* = 93) mice were used. This mouse strain was selected as per recommendations made at the Banbury Conference on Genetic Background in Mice to facilitate the comparison of results between experiments and among laboratories; this strain is often selected for excellent behavioral characteristics, and is a common genetic background of targeted mutations (Silva et al. 1997). Mice were weaned at 3 weeks of age and group housed (2–5 mice per same sex cage) with unrestricted access to food and water. The animal colony was maintained on a 14:10-h light/dark schedule and all testing occurred during the light phase. Mice were at least 10 weeks old and handled for 3 days (1 min/day) prior to testing. All 184 mice were used for fear conditioning; of these mice, 45 (24 males and 21 females) were used 6 weeks later for tests of behavioral sensitization, conditioned place preference, and conditioned responding, and 79 (33 males and 46 females) were used 8 weeks later for the forced swim test. Cages of mice were randomly re-assigned to these additional experiments. All animal care and experimental procedures were approved by the UCSD IACUC and compliant with the NRC Guide for the Care and Use of Laboratory Animals.

### Drugs

3,4-MDMA HCl (CAS No. 64057-70-1; Cayman Chemical, Ann Arbor, MI, USA) was dissolved in 0.9 % physiological saline and given intraperitoneally (i.p.) in a volume of 10 mL/kg. A range of MDMA doses were selected: 0, 0.01, 0.05, 0.1, 0.5, 1, 3, and 10 mg/kg (salt weight).

### Fear conditioning

The VideoFreeze system (Med Associates Inc., St. Albans, VT, USA) and fear conditioning protocol were used as described previously (Anagnostaras et al. 2000, 2010; Carmack et al. 2014; Pantoni et al. 2020; Shuman et al. 2009; Wood and Anagnostaras 2011). Four mice were tested concurrently in individual conditioning chambers (32 × 25 × 25 cm) that consisted of stainless-steel sidewalls and rod floors, white acrylic back walls, and clear polycarbonate front and top walls. Each chamber was transformed across multiple sensory dimensions to create two distinct contexts: a training context, which was used for training and context testing, and an alternate context, which was used for tone testing. For the training context, chambers were cleaned and scented with 7 % isopropanol, and illuminated with moderate (80 lx) white light and near-infrared light (980 nm). For the alternate context, chambers were outfitted with a black plastic, triangular teepee and white acrylic floors, cleaned and scented with a 5 % vinegar solution, and illuminated with only near-infrared light to create a dark environment. VideoFreeze software (Med Associates Inc.) used digital video to score freezing behavior and locomotor activity (Anagnostaras et al. 2010).

A total of 184 mice were randomly assigned to groups by dose of MDMA administered: 0 (*n* = 35), 0.01 (*n* = 20), 0.05 (*n* = 20), 0.1 (*n* = 30), 0.5 (*n* = 20), 1 (*n* = 20), 3 (*n* = 20), or 10 (*n* = 19) mg/kg. Groups were counterbalanced by sex and conditioning chamber. Mice were given an injection of MDMA or saline 30 min before a 10-min training session. A delay of 30 min was selected due to its temporal proximity to peak drug exposure, the first instance of locomotor activity (from pilot work in our lab), core temperature, and behavioral effects following intraperitoneal MDMA in mice (Fantegrossi et al. 2008; for review, see Pantoni and Anagnostaras 2019). Training began with a 3-min baseline period followed by a single tone-shock pairing, which consisted of a 30-s pure tone (2.8 kHz, 85 dBA) presented through a speaker in the chamber sidewall that co-terminated with a 2-s scrambled, AC constant current footshock (0.75 mA, RMS) delivered through the rod floors. Ninety seconds after the tone-shock pairing, mice underwent a 5-min post-shock test. Locomotor activity during the baseline period and during the footshock was used to measure on-drug baseline locomotion and shock reactivity, respectively, while freezing behavior during the post-shock test was used to measure on-drug short-term memory.

Seven days after training, mice were returned to the training context, off drug, for a 5-min context test. Freezing behavior during the test was used to measure long-term context memory. One day after context testing, mice were brought to the alternate context, off drug, for a 5-min tone test. Tone testing consisted of a 2-min baseline period, followed by the presentation of 3, 30-s tones identical to the training tone each separated by 30s. Freezing behavior during the tone presentations was used to measure long-term tone memory.

### Behavioral sensitization, conditioned place preference, and conditioned responding

Eight mice were tested concurrently in individual place preference chambers (Med Associates Inc., St. Albans, VT, USA) as described previously (Carmack et al. 2013, 2014; Pantoni et al. 2020). Each chamber (43 × 43 × 31 cm) consisted of two sides—a drug-paired side and an unpaired side—separated by a black wall with a removable insert. The two sides were visually and tactilely distinct as they differed by flooring (stainless steel rods or wire mesh) and walls (white and decorated with stickers or undecorated clear polycarbonate). Chambers were counterbalanced by flooring and wall combinations and by paired versus unpaired side assignments. Each chamber was cleaned with 10 % glass cleaner (Zep Inc., Atlanta, GA, USA) between trials. Activity Monitor software (Med Associates Inc.) used the interruption of infrared beams to identify mouse position and score locomotion (distance), stereotypy (counts), and verticality (counts).

Forty-five mice were randomly assigned to new groups by dose of MDMA administered: 0 (*n* = 12), 0.1 (*n* = 10), 1 (*n* = 11), or 10 (*n* = 12) mg/kg. These doses were selected to include doses that consistently had no effects in the fear conditioning experiment (0.1 and 1 mg/kg) and a dose that consistently impaired memory in the fear conditioning experiment (10 mg/kg). Groups were counterbalanced by sex and testing chamber. Mice were habituated to the testing chamber, off drug, for 30 min per side per day for 2 consecutive days prior to training (with the order of side placement counterbalanced). Four days after habituation, mice were trained every other day for a total of 7 days. On each training day, mice were injected with saline before being placed into the unpaired side for 15 min, then injected with MDMA before being placed into the paired side for 15 min. Locomotor, stereotyped, and vertical activity on the paired side was scored and behavioral sensitization was calculated as the difference between average activity on day 7 versus day 1.

Twenty-four hours after the last training day, mice were tested off drug for conditioned place preference. The inserts that previously separated the two sides of the chambers were removed. Mice were placed into the entryway between the two sides of the chamber (with the direction of entry counterbalanced) and allowed access to both sides for 15 min. Locomotor activity and time spent on each side was scored and place preference was calculated as the difference between responses on the paired side versus the unpaired side.

Forty-eight hours after the last training day, mice received two back-to-back challenge tests: one with saline and one with a high dose of MDMA (10 mg/kg). Mice were injected with saline and immediately placed into the paired side for 15 min and then removed and injected with 10 mg/kg MDMA and immediately returned to the paired side for 45 min. Locomotor, stereotyped, and vertical activity was scored to evaluate the presence of conditioned responding to the drug-paired side (saline challenge) and/or sensitized responding to the high dose of 10 mg/kg MDMA (high dose challenge). One mouse trained with 10 mg/kg MDMA died during the high dose challenge and its data was excluded from that test only.

### Forced swim test

The forced swim test procedure was adapted from existing protocols (Can et al. 2012; Castagné et al. 2011; Porsolt et al. 2001; Yankelevitch-Yahav et al. 2015). Five mice were tested concurrently in individual cylindrical beaker-like glass tanks (10 cm diameter × 24 cm height) that were visually separated by white opaque acrylic dividers. Each tank was filled with water (24 ± 0.5 °C) to a depth of 15 cm. Mice were tested in moderate light (approximately 80 lx) and immobility was measured using an HD USB video camera and behavioral tracking software (ANY-Maze, Wood Dale, IL, USA; minimum immobility time = 2000 ms, immobility sensitivity = 75 %).

Seventy-nine mice were randomly assigned to new groups by dose of MDMA administered: 0 (*n* = 14), 0.1 (*n* = 13), 0.5 (*n* = 13), 1 (*n* = 13), 3 (*n* = 13), or 10 (*n* = 13) mg/kg. Groups were counterbalanced by sex and testing tank. Mice were given an injection of MDMA or saline 30 min before testing. Mice were placed into the water for a 6-min test and the time spent immobile was scored during the last 4 min to evaluate potential antidepressant effects (reduced immobility).

### Statistical analyses

**Fig. 1.**
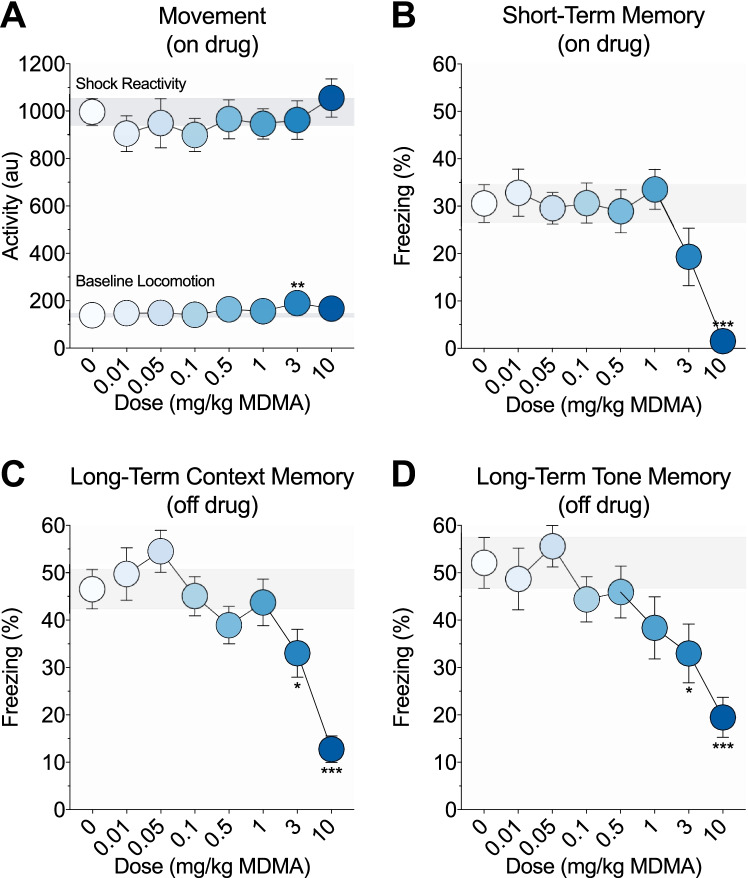
Effects of MDMA on fear learning and memory. **a** On-drug activity during the 3-min training baseline period and the 2-s footshock. Mice given 3 mg/kg MDMA showed increased baseline locomotion relative to saline controls. There were no group differences in shock reactivity. **b** Short-term memory as measured by percent freezing during the on-drug post-shock test. Mice given 10 mg/kg MDMA showed impaired short-term memory relative to saline controls. **c** Long-term context memory as measured by percent freezing during the off-drug context test, 1 week after training. Mice previously given 3 or 10 mg/kg MDMA showed impaired long-term context memory relative to saline controls. **d** Long-term tone memory as measured by percent freezing during the off-drug tone test, 1 day after context testing. Mice previously given 3 or 10 mg/kg MDMA showed impaired long-term tone memory relative to saline controls. Each point represents the mean ± 1 standard error. The gray bar indicates standard error range for the comparison saline control group. Asterisks identify significant comparisons against the saline control group using Fisher’s LSD (*P < 0.05, **P < 0.01, and ***P< 0.001)

**Fig. 2.**
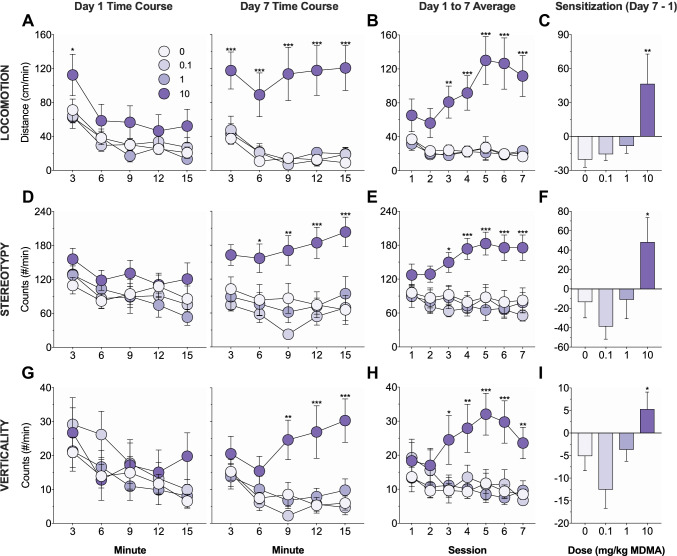
Effects of MDMA on behavioral sensitization. Mice were trained for 7 days and locomotion (**a**–**c**), stereotypy (**d**–**f**), and verticality (**g**–**i**) on the drug-paired side were measured. **a**, **d**, **g** Time course of activity on day 1 (left) and day 7 (right) of training. There were no group differences on day 1, but on day 7, mice receiving 10 mg/kg MDMA exhibited increased locomotion (**a**), stereotypy (**d**), and verticality (**g**) relative to saline controls. **b**, **e**, **h** Average activity on each of the seven days of training. Mice receiving 10 mg/kg MDMA exhibited increased locomotion (**b**), stereotypy (**e**), and verticality (**h**) relative to saline controls from day 3 to day 7. **c**, **f**, **i** Development of sensitization as measured by the difference in average activity on day 7 versus day 1. Mice receiving 10 mg/kg MDMA exhibited a greater increase in locomotion (**c**), stereotypy (**f**), and verticality (**i**) from day 1 to day 7 relative to saline controls. Asterisks identify significant comparisons against the saline control group at the same time point

**Fig. 3.**
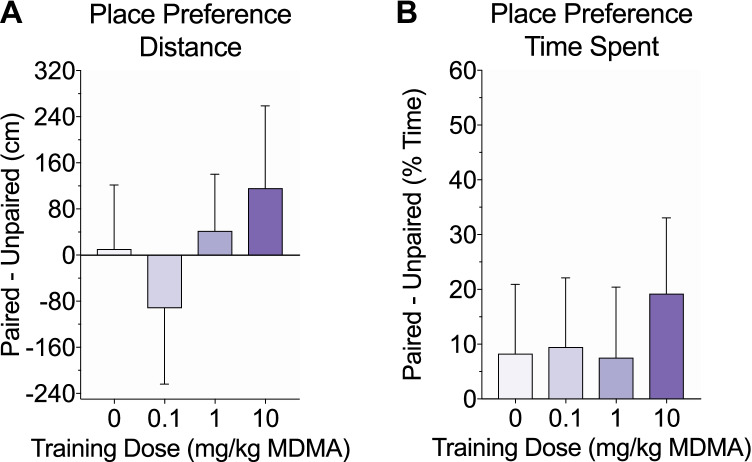
Effects of MDMA on conditioned place preference. Following 7 days of training, mice were tested off drug for place preference, which was measured by the difference in distance traveled (**a**) and time spent (**b**) on the drug-paired side versus the unpaired side. There were no significant group differences and none of the groups exhibited a significant preference for either side

**Fig. 4.**
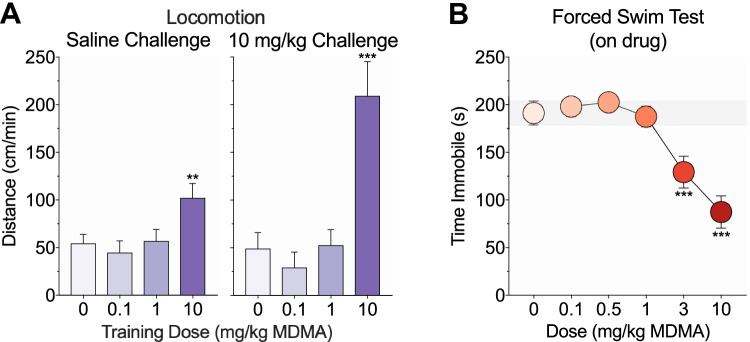
Effects of MDMA on conditioned and sensitized responding, and depressive-like behavior. **a** Following training and place preference testing, mice underwent saline (left) and high-dose MDMA (right) challenge tests on the paired side and locomotion was scored to evaluate conditioned and sensitized responding, respectively. Mice trained with 10 mg/kg MDMA showed increased locomotion relative to saline controls following both challenge injections. **b** A separate cohort of mice underwent a 6-min on-drug forced swim test and time spent immobile was measured during the last 4 min of testing. Mice given 3 or 10 mg/kg MDMA exhibited reduced immobility relative to saline controls

Data were analyzed using univariate or multivariate analyses of variance (ANOVAs/MANOVAs) to identify overall group differences. MANOVAs were used for repeated measures and within-subjects data (Fig. 2a, b, d, e, g, h). ANOVAs were used for individual pieces of between-subjects data (Figs. 1, 2c, f, i, 3, 4). Post hoc comparisons were performed following significant group differences using Fisher’s least significant difference (LSD) tests against the saline control group. Except for Supplementary Fig. 1, data from male and female mice were merged as we found no other statistically significant sex differences that meaningfully influenced these findings (p values > 0.05).

## Results

### Fear conditioning

The effects of MDMA (0–10 mg/kg, i.p.) on fear learning and memory were examined using Pavlovian fear conditioning. Mice were trained on drug with a single tone-shock pairing. Freezing was scored during an on-drug post-shock test and 1 week later during an off-drug context test and an off-drug tone test to evaluate short- and long-term fear memory. MDMA weakly dose-dependently modulated locomotor activity during the training baseline period (F(7, 176) = 2.08, *p* = 0.05; Fig. 1a, lower line). Only mice given 3 mg/kg MDMA showed significantly increased baseline locomotion compared to saline controls (*p* = 0.001; all other *p* values > 0.07). The shock elicited a large activity burst unconditioned response that did not significantly differ between groups (F(7, 176) = 0.43, *p* = 0.88; Fig. 1a, upper line). MDMA dose-dependently modulated freezing during the on-drug post-shock (F(7, 176) = 5.24, *p* < 0.001; Fig. 1b), off-drug context (F(7, 176) = 7.17, *p* < 0.001; Fig. 1c), and off-drug tone (F(7, 176) = 3.98, *p* < 0.001; Fig. 1d) tests. Compared to saline controls, only mice given 10 mg/kg MDMA exhibited reduced freezing during the post-shock test (*p* < 0.001; all other *p* values > 0.05), and only mice previously given 3 or 10 mg/kg MDMA exhibited reduced freezing during the context (*p* values ≤ 0.03; all other *p* values > 0.1) and tone (*p* values ≤ 0.01; all other *p* values > 0.06) tests.

### Behavioral sensitization, conditioned place preference, and conditioned responding

The effects of MDMA (0–10 mg/kg, i.p.) on addiction-related behaviors were examined using tests of behavioral sensitization, conditioned place preference, and conditioned responding. Mice were trained for 7 days in a two-sided chamber; on each day, mice were injected with saline and placed into the unpaired side and then injected with MDMA and placed into the paired side. Locomotor, stereotyped, and vertical activity on the drug-paired side was measured. Significant group differences in activity were not observed on day 1 (locomotion, F(3, 41) = 1.77, *p* = 0.17; stereotypy, F(3, 41) = 1.06, *p* = 0.38; verticality, F(3, 41) = 0.43, *p* = 0.74; Fig. 2a, d, g, left), but were observed on day 7 (locomotion, F(3, 41) = 11.85, *p* < 0.001; stereotypy, F(3, 41) = 7.54, *p* < 0.001; verticality, F(3, 41) = 6.82, *p* < 0.001; Fig. 2a, d, g, right). Compared to saline controls, only mice receiving 10 mg/kg MDMA showed significantly increased locomotor (*p* < 0.001; all other *p* values > 0.7), stereotyped (*p* = 0.001; all other *p* values > 0.3), and vertical (*p* < 0.001; all other *p* values > 0.6) activity on day 7.

There were also significant main effects of group (locomotion, F(3, 41) = 11.23, *p* < 0.001; stereotypy, F(3, 41) = 7.21, *p* < 0.001; verticality, F(3, 41) = 3.64, *p* = 0.02) and group-by-day interactions (locomotion, F(18, 246) = 3.6, *p* < 0.001; stereotypy, F(18, 246) = 2.51, *p* < 0.001; verticality, F(18, 246) = 3.51, *p* < 0.001) on average daily activity across the seven days of training (Fig. 2b, e, h). Compared to saline controls, only mice receiving 10 mg/kg MDMA showed significantly increased locomotor (*p* < 0.001; all other *p* values > 0.8), stereotyped (*p* = 0.001; all other *p* values > 0.5), and vertical (*p* = 0.007; all other *p* values > 0.6) activity, and these effects were observed on the last 5 days (locomotion, *p* values ≤ 0.002; stereotypy, *p* values ≤ 0.02; verticality, *p* values ≤ 0.01) but not the first 2 days of training (locomotion, *p* values > 0.07; stereotypy, *p* values > 0.09; verticality, *p* values > 0.1). Lastly, there were significant group differences in the development of sensitization as measured by the difference in average activity on day 7 versus day 1 (locomotion, F(3, 41) = 4.42, *p* = 0.009; stereotypy, F(3, 41) = 3.57, *p* = 0.02; verticality, F(3, 41) = 4.32, *p* = 0.01; Fig. 2c, f, i). Only mice receiving 10 mg/kg MDMA exhibited a significant increase in locomotor (*p* = 0.002; all other *p* values > 0.5), stereotyped (*p* = 0.03; all other *p* values > 0.3), and vertical (*p* = 0.04; all other *p* values > 0.1) activity from day 1 to day 7 when compared to saline controls.

Twenty-four hours after the last training day, mice were tested off drug for conditioned place preference. Mice were allowed free access to both sides and place preference was measured by the difference in distance traveled and time spent on the drug-paired side versus the unpaired side. There were no significant group differences in distance traveled (F(3, 41) = 0.48, *p* = 0.7; Fig. 3a) or time spent (F(3, 41) = 0.18, *p* = 0.91; Fig. 3b) between sides. Additionally, none of the groups exhibited place preference in locomotor activity (one sample two-tailed *t*-test against hypothesized *μ* = 0; t(9–11) values < 1, *p* values > 0.4) or time spent (t(9–11) values < 1.4, *p* values > 1).

Forty-eight hours after the last training day, mice were challenged with saline and then a high dose of MDMA (10 mg/kg) on the paired side. Locomotor, stereotyped, and vertical activity in response to the saline challenge and the high-dose challenge was scored to evaluate conditioned and sensitized responding, respectively. There were significant group differences in locomotion following the saline (F(3, 41) = 4.31, *p* = 0.01; Fig. 4a, left) and high-dose MDMA (F(3, 40) = 13.14, *p* < 0.001; Fig. 4b, right) challenges. Compared to saline controls, only mice trained with 10 mg/kg MDMA exhibited a CR as measured by increased locomotion following the saline challenge (*p* = 0.008; all other *p* values > 0.5) or sensitization as measured by increased locomotion following the high dose MDMA challenge (*p* < 0.001; all other *p* values > 0.5). The same pattern of effects was observed for stereotypy (group differences, *p* values ≤ 0.02; 10 mg/kg versus saline, *p* values ≤ 0.03) but not verticality (no group differences, *p* values > 0.2) (data not depicted).

### Forced swim test

The effects of MDMA (0–10 mg/kg, i.p.) on depressive-like behavior were examined using the forced swim test. Mice underwent a 6-min on-drug test and time spent immobile was scored during the last 4 min of testing. MDMA dose-dependently modulated immobility (F(5, 73) = 13.13, *p* < 0.001; Fig. 4b). Only mice given 3 or 10 mg/kg MDMA exhibited reduced immobility relative to saline controls (*p* values < 0.001; all other *p* values > 0.5).

## Discussion

The present study provides further evidence for the critical role of dose selection in the behavioral effects of MDMA. Specifically, we found that high doses of MDMA produced fear memory impairments (at 3 and 10 mg/kg), some evidence of an addictive potential (at 10 mg/kg), and antidepressant effects (at 3 and 10 mg/kg), while low doses of MDMA (≤ 1 mg/kg) did not. Frequent high-dose MDMA (≥ 3 mg/kg) should likely be avoided for its amnesic effects and addictive potential but low-dose MDMA, which has been administered in recent clinical studies (approximately 1–2 mg/kg MDMA; for review, see Feduccia et al. 2018), is likely safe in terms of the behaviors analyzed herein. It appears that MDMA has a narrow viable therapeutic window and lowering dose should remain an important consideration in clinical use.

Our earlier systematic review (Pantoni and Anagnostaras 2019) questioned concerns that therapeutic use of MDMA would cause memory problems, as there was no evidence from animal research that MDMA impairs cognition at low, clinically relevant doses (< 3 mg/kg) but results regarding higher doses (≥ 3 mg/kg) were mixed. The present dose-effect analysis provides further evidence that 3 mg/kg MDMA appears to be the threshold for memory impairments. Using a Pavlovian fear conditioning paradigm, 10 mg/kg MDMA impaired short-term memory (on drug), 3 and 10 mg/kg MDMA impaired long-term context and tone memory (off drug), and 0.01 to 1 mg/kg MDMA did not impair memory. These fear memory impairments were not confounded by effects on nociception, as demonstrated by lack of group differences in shock reactivity, nor by effects on locomotor activity, as the short-term memory-impairing dose of 10 mg/kg MDMA had no effect on baseline locomotion and the long-term memory tests were conducted off drug. Our data suggests that high-dose MDMA produces anterograde amnesia because short- and long-term fear memory impairments were comparable. Further investigation is required to isolate the nature of the observed fear memory impairments, which could be caused by failures to encode, consolidate, or retrieve the memory, associate the memory with an aversive outcome, discriminate the context or tone from other places the animal has been or sounds they have heard, or engage defensive behavior to diffuse predictors of shock (Gerlai 2001; Maren 2008).

We did not detect any MDMA-induced fear memory enhancements even though psychostimulants enhance memory at low, clinically relevant doses (Carmack et al. 2014; Shuman et al. 2009; Wood and Anagnostaras 2009; Wood et al. 2007) and there is sparse evidence that MDMA may sometimes enhance cognition (for review, see Pantoni and Anagnostaras 2019). Instead, MDMA produced dose-dependent effects that were similar to that of the selective serotonin reuptake inhibitor citalopram (i.e., no effects at low, clinically relevant doses; impairments at high doses; Carmack et al. 2014). It is possible that MDMA does not act strongly enough at DAT and NET to enhance fear memory or that drug action at SERT interferes with fear memory enhancement. Enhanced memory reconsolidation and fear extinction has been proposed as a potential therapeutic mechanism of MDMA-assisted psychotherapy for post-traumatic stress disorder (Feduccia and Mithoefer 2018). While we did not detect changes in fear learning at low, clinically relevant doses of MDMA, high-dose MDMA (7.8 mg/kg) has been reported to enhance fear memory extinction (Young et al. 2015, 2017), and further research should investigate the effects of low-dose MDMA on fear extinction.

The addictive potential of high-dose psychostimulants is reflected in their propensity to elicit dramatic locomotor stimulation, behavioral sensitization, conditioned place preference, and conditioned responding (Anagnostaras and Robinson 1996; Anagnostaras et al. 2002; Carmack et al. 2014, 2017; Robinson and Berridge 1993, 2003, 2008; Shuman et al. 2012). We found that treatment with low, clinically relevant doses of 0.01 and 1 mg/kg MDMA did not lead to any addiction-related behaviors, even when tested with the 10 mg/kg MDMA high-dose challenge. Treatment with a high, memory-impairing dose of 10 mg/kg MDMA did lead to behavioral sensitization and conditioned responding, but not acute locomotor stimulation or conditioned place preference. Other drug-pairing procedures similarly have been found to occasion behavioral sensitization or conditioned responding in the absence of conditioned place preference (Brown and Fibiger 1993; Carmack et al. 2013; Hemby et al. 1992; Rowlett et al. 1994; Seymour and Wagner 2008). Furthermore, we observed interesting sex differences in the effects of 10 mg/kg MDMA on acute locomotor activity, as only females showed increased locomotion starting on the first day of training (see Supplementary Results). This may be related to findings that females are more sensitive than males to the psychological effects of MDMA (for review, see Allott and Redman 2007 and Liechti et al. 2001). However, both sexes similarly developed sensitization.

Our observations are inconsistent with previous reports of MDMA-induced place preference in mice at high doses of about 10 m/kg (for example, see Robledo et al. 2004; Salzmann et al. 2003). It is possible that the weak place preference observed in our 10 mg/kg MDMA group was non-significant due to insufficient statistical power. It has also been reported that MDMA does not induce place preference in group-housed animals (Meyer et al. 2002). Still, even if this effect were significant, its magnitude (difference in time spent on the drug-paired versus unpaired side = 19.2 %) is far weaker than that observed in our previous psychostimulant studies (around 50 %; Carmack et al. 2014; Shuman et al. 2012). Other head-to-head comparisons of psychostimulants versus MDMA have similarly found that rodents treated with methamphetamine and methylphenidate (Mori et al. 2021) as well as amphetamine (Meyer et al. 2002) exhibit significant place preference but those treated with comparable doses of MDMA do not. Altogether, our findings suggest that repeated use of MDMA at high (but not low) doses may lead to compulsive drug taking and drug-cue elicited craving, although MDMA may be less rewarding and less likely to provoke drug seeking than psychostimulants and other drugs that induce strong conditioned place preference (for reviews, see Carmack et al. 2017 and Tzschentke 2007). Indeed, the level of locomotor activity seen after MDMA administration seems to suggest that even at high doses, it is only a very modest psychomotor stimulant.

There are opposing views regarding how MDMA modulates depressive symptoms—one view holds that MDMA exacerbates mood problems including depression (for review, see Morgan 2000), while the other holds that MDMA has antidepressant properties that are implicated in its therapeutic effects (Thal and Lommen 2018; Yazar-Klosinski and Mithoefer 2017). Recent clinical studies report both depression symptom improvement as a secondary outcome and depressed mood as a treatment-emergent adverse event following MDMA-assisted psychotherapy (Mithoefer et al. 2019). Using the forced swim test, we detected acute MDMA-induced antidepressant effects at high, memory-impairing doses of 3 and 10 mg/kg but not at lower doses of 0.1, 0.5, and 1 mg/kg. Drugs that induce acute locomotor stimulation can lead to a false positive result in the forced swim test (Porsolt et al. 1978). This is a common concern with psychostimulants; however, there is clinical data suggesting that psychostimulants do indeed alleviate depressive symptoms and thus the term “false positive” may be misleading (Candy et al. 2008; Castagné et al. 2011). It is unlikely that locomotor stimulation was responsible for decreased immobility in the present study as we found little evidence that a single dose of 3 or 10 mg/kg MDMA acutely stimulates locomotor activity when averaged across both sexes. Since we found no acute antidepressant effects at low, clinically relevant doses, it is possible that low-dose MDMA requires chronic administration to reduce depressive-like behavior as do low-dose selective serotonin reuptake inhibitor, norepinephrine reuptake inhibitor, tricyclic, and monoamine oxidase inhibitor antidepressants (Cryan et al. 2005a, b; Detke et al. 1997; Vázquez-Palacios et al. 2004). Low, non-amnesic doses of MDMA may also have other therapeutic effects such as increased sociality or openness that facilitate the clinical improvements observed following MDMA-assisted psychotherapy by, for example, improving the doctor-patient therapeutic alliance (Heifets and Malenka 2016; Wagner et al. 2017).

There is increasing evidence that the therapeutic effects of MDMA are mediated by the serotonergic system whereas its addiction-related effects are primarily mediated by the dopaminergic system. Young et al. (2017) demonstrated that the action of MDMA at SERT and subsequent 5-HT2A receptor activation plays an important role in its enhancement of fear memory extinction. Similarly, Heifets et al. (2019) demonstrated that the action of MDMA at SERT and subsequent 5-HT1B receptor activation within the nucleus accumbens is necessary and sufficient for its prosocial effects, whereas MDMA binding at DAT and the consequent increase in DA release is required for its rewarding effects. Risbrough et al. (2006) revealed that the DA receptor subtypes have differential modulatory roles in MDMA-induced hyperactivity; specifically, D1 receptor activation modifies the type of activity (linear versus circumscribed) whereas D2 receptor activation contributes to repetitive circling behavior. It is generally accepted that DAT is responsible for the addictive properties of MDMA, although there is some evidence that SERT may play a lesser role (Trigo et al. 2007). In any case, the evidence is that MDMA use does not often result in addiction (Degenhardt et al. 2010).

The effects of MDMA on the serotonergic versus dopaminergic systems are also dose-dependent. At low doses (< 3 mg/kg), MDMA stimulates 5-HT release and little to no DA release, whereas at high doses (≥ 3 mg/kg), MDMA stimulates both 5-HT and DA release (Baumann et al. 2005, 2007; Kankaanpää et al. 1998). In accordance with these findings, we detected MDMA-induced addiction-related behaviors at high doses that correlate with substantial DA release. Additional evidence suggests that the R(-) enantiomer of MDMA retains the prosocial and fear extinction-enhancing effects but possibly not the abuse liability of racemic MDMA because of its significantly decreased potency as a DA releaser (Pitts et al. 2018). It is plausible that low-dose racemic MDMA or another drug that preferentially induces 5-HT release may promote prosocial behavior and have a significantly reduced abuse liability.

Our findings suggest that therapeutic use of MDMA below 3 mg/kg is less likely to produce significant adverse cognitive effects. While psychostimulants have the potential for addiction and toxicity at high doses, they are effective and safe cognitive enhancers that are prescribed at low doses for extended periods of time (for review, see Wood et al. 2014). Similarly, MDMA is showing promise as a psychotherapeutic, and low doses seem to pose little risk of memory impairments, addiction, or depressed mood. It is important to note that the dose threshold for potential memory impairments and addiction (3 mg/kg MDMA) is close to the doses used in recent clinical studies (approximately 1–2 mg/kg MDMA; for review, see Feduccia et al. 2018) and this may limit therapeutic viability. Future studies should consider exploring ultra-low doses of MDMA (< 1 mg/kg), which, like psychostimulants (e.g., Wood and Anagnostaras 2009), may be even safer and as effective compared to low doses (1–2 mg/kg). In all, we believe that the potential adverse effects of MDMA must be considered within the framework of its therapeutic application, with a particular orientation to the use of low doses.

## Supplementary information

ESM 1 (PDF 138 kb)

## Footnotes

1 In this article, we refer to “addictive potential” rather than “abuse potential” because even acute recreational use of MDMA is considered abuse. The existence of an illicit market means that, at present, any use of MDMA outside of a clinical trial is considered abuse. Rather, we are specifically referring to the potential to develop addiction.
