# Correction to: MDMA and memory, addiction, and depression: dose-effect analysis

**DOI:** 10.1007/s00213-022-06126-4

**Published:** 2022-04-19

**Authors:** Madeline M. Pantoni, Jinah L. Kim, Kaitlin R. Van Alstyne, Stephan G. Anagnostaras

**Affiliations:** 1 Molecular Cognition Laboratory, Department of Psychology, University of California San Diego, La Jolla, CA USA; 2 Department of Psychiatry and Behavioral Sciences, University of California San Francisco, San Francisco, CA USA; 3 Molecular Cognition Laboratory, Program in Neurosciences, University of California San Diego, La Jolla, CA USA

**Correction to: Psychopharmacology**

10.1007/s00213-022-06086-9

The PDF version of this originally published article was the uncorrected proof; it has now been replaced by the corrected version. The publisher apologizes for the inconvenience caused.

The original article has been corrected.

