# Supplementary material for: MDMA and memory, addiction, and depression: dose-effect analysis
Source: Psychopharmacology (Berl). 2022 Feb 18;239(3):935–49. doi: 10.1007/s00213-022-06086-9 (PMC8891111; doi:10.1007/s00213-022-06086-9)
Supplement: Supplementary file 1 — (PDF 138 kb) [file 213_2022_6086_MOESM1_ESM.pdf]

Article Title: MDMA and memory, addiction, and depression: dose-effect analysis

Journal Name: Psychopharmacology

Author Names: Madeline M. Pantoni, Jinah L. Kim, Kaitlin R. Van Alstyne, and Stephan G. Anagnostaras

Corresponding Author: Madeline M. Pantoni**;** Molecular Cognition Laboratory, Department of Psychology, University of California San Diego, La Jolla, California, United States; maddie.pantoni@gmail.com

Supplementary Results

We found statistically significant sex differences in the effects of MDMA on locomotor activity during training. There was a main effect of sex that trended towards significance [$F(1, 37) = 3.06$, $p = 0.09$] and a significant group-by-sex interaction [$F(3, 37) = 9.99$, $p < 0.001$] on Day 1 (**Supplementary Figure 1A, left**). There was also a significant main effect of sex [$F(1, 37) = 5.76$, $p = 0.02$] and a significant group-by-sex interaction [$F(3, 37) = 3.27$, $p = 0.03$] on Day 7 (**Supplementary Figure 1A, right**). Sex differences were observed only in mice receiving 10 mg/kg MDMA (Day 1: $p < 0.001$, all other p values > 0.2; Day 7: $p < 0.001$, all other p values > 0.3). Compared to saline controls of the same sex, female ($p < 0.001$) but not male ($p = 0.17$) mice receiving 10 mg/kg MDMA showed significantly increased locomotion on Day 1, and both female ($p < 0.001$) and male ($p = 0.01$) mice receiving 10 mg/kg MDMA showed significantly increased locomotion on Day 7.

There was also a significant main effect of sex [$F(1, 37) = 7.61$, $p = 0.009$] and a significant group by-sex interaction [$F(3, 37) = 11.8$, $p < 0.001$] on average daily locomotion across the seven days of training (**Supplementary Figure 1B**). Sex differences were observed only in mice receiving 10 mg/kg MDMA ($p < 0.001$; all other p values > 0.6). Compared to saline controls of the same sex, female ($p < 0.001$) but not male ($p = 0.09$) mice receiving 10 mg/kg MDMA showed significantly increased locomotion across the seven days of training. In female mice, there was a significant main effect of group [$F(3, 17) = 39.12$, $p < 0.001$] and a significant group-by-day interaction [$F(18, 102) = 2.07$, $p = 0.01$]. Compared to female saline controls, only female mice receiving 10 mg/kg showed significantly increased locomotion ($p < 0.001$; all other p values > 0.8) and this effect was observed on all seven days of training (p values ≤ 0.002). In male mice, there was no significant main effect of group [$F(3, 20) = 1.63$, $p = 0.21$] but there was a significant group-by-day interaction [$F(18, 120) = 2.02$, $p = 0.01$]. Compared to male saline controls, only male mice receiving 10 mg/kg showed significantly increased locomotion on the last three days (p values ≤ 0.04) but not the first four days (p values > 0.2) of training. Despite significant sex differences in the acute effects of MDMA on locomotion, no main effect of sex [$F(1, 37) = 0.71$, $p = 0.41$] or group-by-sex interaction [$F(3, 37) = 0.19$, $p = 0.9$] was observed for the development of sensitization as measured by the difference in average locomotion on Day 7 versus Day 1 (**Supplementary Figure 1C**).

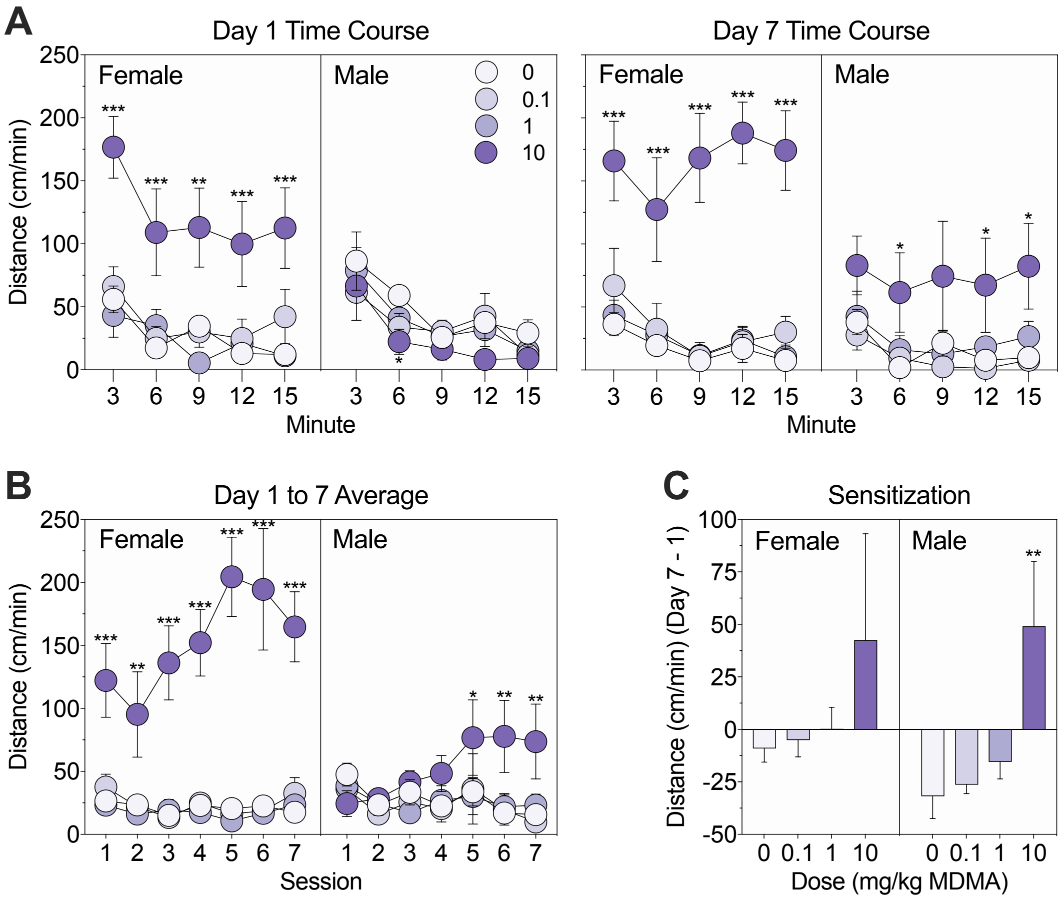

Supplementary Figure 1 Sex differences in effects of MDMA on locomotion. Data from Figure 2 divided by female **(left)** and male **(right)** mice. **(A)** Time course of locomotion on Day 1 **(left)** and Day 7 **(right)** of training. Female mice receiving 10 mg/kg MDMA exhibited increased locomotion relative to female saline controls on Days 1 and 7. Male mice receiving 10 mg/kg MDMA exhibited increased locomotion relative to male saline controls on Day 7 only. **(B)** Average locomotion on each of the seven days of training. Female mice receiving 10 mg/kg MDMA exhibited increased locomotion relative to female saline controls on all seven days. Male mice receiving 10 mg/kg MDMA exhibited increased locomotion relative to male saline controls from Day 5 to Day 7 only. **(C)** Development of sensitization as measured by the difference in average locomotion on Day 7 versus Day 1. There was no main effect of sex or group-by-sex interaction. Asterisks identify significant comparisons against the saline control group of the same sex and at the same time point.
